# pH-sensitive fibronectin nanogels combined with UTMD for anti-atherosclerosis treatment through anti-inflammatory and antioxidant effects

**DOI:** 10.1016/j.mtbio.2025.102044

**Published:** 2025-07-08

**Authors:** Mengjiao Zhang, Xianghao Xiao, Xinyi Li, Xiangyang Shi, Zhaojun Li

**Affiliations:** a Department of Ultrasound, Shanghai General Hospital, Shanghai Jiao Tong University School of Medicine, Shanghai, 200080, PR China; b School of Medical Imaging, Shandong Second Medical University, Weifang, Shandong, 261053, PR China; c Department of Ultrasound, Jiading Branch of Shanghai General Hospital, Shanghai Jiao Tong University School of Medicine, Shanghai, 201803, PR China; d College of Biological Science & Medical Engineering, Donghua University, Shanghai, 201620, PR China; e School of Life Sciences, Hubei University, Hubei, 430062, PR China

**Keywords:** Atherosclerosis, Fibronectin, Nanogels, Ultrasound-targeted microbubble destruction

## Abstract

The reactive oxygen species (ROS) and inflammatory factors secreted by macrophages play pivotal roles in all stages of atherosclerotic progression. Achieving targeted plaque localization while simultaneously reducing ROS levels to exert both anti-inflammatory and antioxidant effects remains a significant challenge. In this work, we developed a pH sensitive curcumin (Cur)-loaded fibronectin (FN) nanogels, combined with ultrasound-targeted microbubble destruction (UTMD) to enhance therapeutic efficacy via cavitation effects. The FN/FBA-PEG-FBA@Cur (denoted as FNC) nanogels demonstrated a targeted effect on atherosclerosis sites, attributed to the strong affinity between the RGD sequence on FN and the integrins highly expressed on the surface of plaque cells. Moreover, the Schiff base linkages in the nanogels respond to the acidic microenvironment within atherosclerosis plaques, leading to the controlled Cur release, which enable anti-inflammatory and antioxidant activity to attenuate plaque progression. Both *in vitro* and *in vivo* experiments confirmed enhanced suppression of plaque progression following treatments with FNC nanogels under UTMD. In summary, the FN nanogels capable of targeting to atherosclerosis plaques offers a promising therapeutic approach for atherosclerosis.

## Graphical abstract

**Figure:**
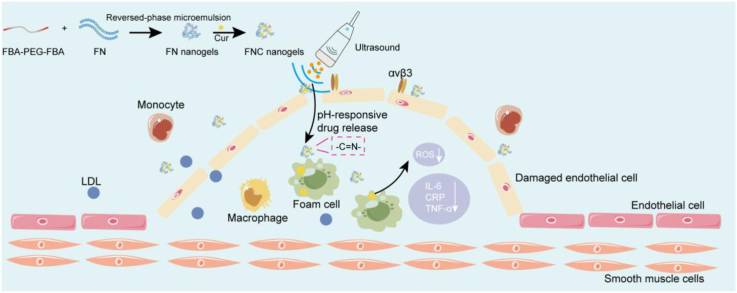

## 1 Introduction

Cardiovascular disease (CVD) remains one of the leading causes of death worldwide, accounting for nearly one-third of all annual mortalities, with atherosclerosis being a primary underlying cause [[1], [2], [3]]. Inflammation plays a central role in the pathology of atherosclerosis, with oxidative stress and pro-inflammatory factors serving as primary drivers of disease progression [[4], [5], [6]]. Excessive reactive oxygen species (ROS) damage vascular endothelial cells [7], increasing endothelial permeability and facilitating the entry of low-density lipoprotein (LDL) into the vascular intima [8,9]. ROS also oxidized LDL to form oxidized LDL (ox-LDL), which not only prompts macrophages to recognize and engulf it, forming foam cells, but also further stimulates local inflammation [10]. Damaged endothelial cells, along with immune cells like macrophages, secrete various pro-inflammatory factors (such as IL-6, TNF-α, and MCP-1), promoting monocyte recruitment and accelerating their polarization into macrophages, thereby amplifying the inflammatory cascade within plaque [[11], [12], [13]]. The current therapeutic paradigm for atherosclerosis continues to be largely confined to pharmacological lipid modulation [14]. It is therefore essential to identify an effective anti-inflammatory and antioxidant therapeutic strategy to inhibit the atherosclerotic cascade.

Fibronectin (FN) is a high-molecular-weight glycoprotein primarily secreted by fibroblasts, endothelial cells, and macrophages [[15], [16], [17], [18]]. Compared to synthetic biomaterials, FN offers excellent biocompatibility, making it an ideal carrier for drug delivery. With multiple domains that bind integrin receptors on cell surfaces, FN facilitates interactions between cells and the extracellular matrix, providing strong targeting capabilities [19,20]. Previous studies have shown that FN directly modulates macrophage behavior by recognizing and activating integrins through the arginine-glycine-aspartic acid (RGD) sequence on its molecular backbone, resulting in effective inflammation targeting [[21], [22], [23]]. Furthermore, FN demonstrates anti-inflammatory and protective effects by inhibiting the nuclear factor-kappa B (NF-κB) pathway in the treatment of various inflammatory diseases, including acute lung injury, Parkinson's disease, and osteoarthritis [[24], [25], [26], [27]]. FN also holds significant potential as a therapeutic protein for inflammatory diseases, as it promotes macrophage polarization toward an anti-inflammatory phenotype and reduces the expression of inflammatory factors [28]. Despite its advantages, free-form FN is prone to degradation in both physiological and culture conditions, has a short half-life in the body, and exhibits diminished adhesion to target cells or tissues. Therefore, it is essential to develop a rational approach to modify FN for improved stability and functionality *in vivo*.

**Scheme 1:**
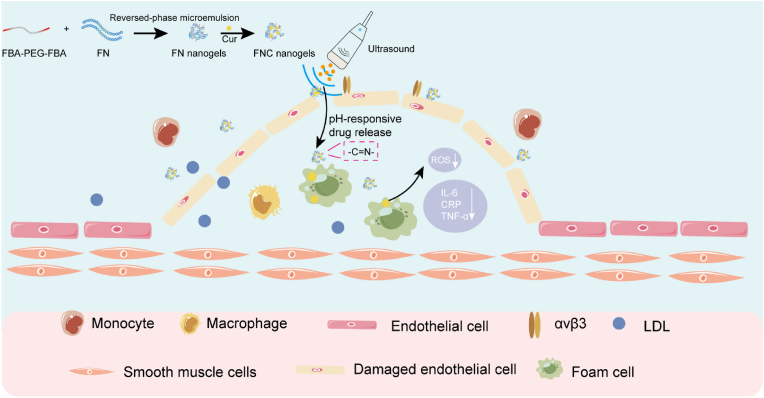
The synthesis scheme of pH-responsive FNC nanogels and their combined mechanism with UTMD to attenuate atherosclerotic progression via dual modulation of plaque inflammation and ROS.

In this study, we utilized FBA-PEG-FBA as a linker to crosslink FN via a pH-sensitive Schiff base, forming nanogels that preserves FN stability without compromising its biological activity. Curcumin (Cur), a potent antioxidant, was encapsulated within the nanogels to enhance its stability and water solubility, thus promoting greater antioxidant efficacy [[29], [30], [31]]. Furthermore, we incorporated ultrasound-targeted microbubble destruction (UTMD)—an ultrasound-triggered effect that enhances tissue permeability through microbubble oscillation—to further improve the localized delivery of nanogels [[31], [32], [33], [34]]. Building on this, the FN nanogels targets plaque sites by binding to αvβ3, which is overexpressed on plaque cells, via its surface RGD sequence. The application of UTMD induces micropores in the plaque endothelium, further accelerating nanogels accumulation. The mildly acidic environment within the plaque triggers the cleavage of the Schiff base, releasing Cur and FN to exert their respective anti-inflammatory and antioxidant effects, thus inhibiting atherosclerotic inflammation (Scheme 1). These FN-based responsive nanogels system presents a promising strategy for the treatment of atherosclerosis.

## 2 Results and discussion

### 2.1 Synthesis and characterization of FNC nanogels

**Fig. 1:**
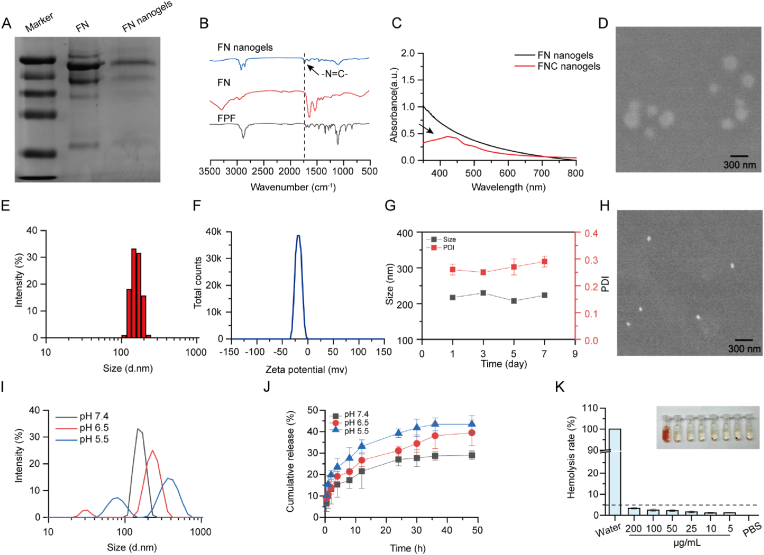
Synthesis and c**haracterization of pH-**s**ensitive,** c**ontrolled-**r**elease, and** h**emocompatible** FNC nanogels. (A) SDS-PAGE analysis of free FN and FN nanogels. (B) FTIR spectra of FN, FBA-PEG-FBA, and FN nanogels, highlighting characteristic group changes. (C) UV–vis spectra of FN nanogels and FNC nanogels. (D) SEM image of FNC nanogels (Scale bar = 300 nm). (E–F) Particle size distribution and zeta potential of FNC nanogels measured by dynamic light scattering (DLS). (G) Hydrodynamic size of FNC nanogels were monitored over one week. Means ± SD, n = 3. (H) SEM image of FNC nanogels under acidic conditions. (I) Size variations of FNC nanogels at different pH values. (J) *In vitro* release profile of Cur from FNC nanogels in PBS under various pH. Means ± SD, n = 3. (K) Hemolysis rate of red blood cells incubated with FNC nanogels at various concentrations, along with images of red blood cell suspensions. Means ± SD, n = 3.

FN nanogels were synthesized using a reverse-phase microemulsion method, with FN as the base material and FBA-PEG-FBA as the cross-linking agent. The amino group of FN can react with the aldehyde group of FBA-PEG-FBA to generate a pH-sensitive Schiff base, which can response to the plaque acid microenvironment. As demonstrated by SDS-PAGE, FN nanogels exhibited protein bands identical to free FN (Fig. 1A), demonstrating that molecular structure of FN remained intact after the reaction. The presence of aldehyde groups was confirmed by Fourier-transform infrared (FTIR) spectroscopy, which identified a characteristic peak of -N=C- at 1659 cm^−1^ (Fig. 1B), proving the present of the crosslinked structure and formation of nanogels. To furtherly combat inflammatory factors and ROS in the microenvironment of atherosclerotic plaques, Cur, as anti-inflammatory agent, was incorporated into the nanogels, resulting in the formation of FN/FBA-PEG-FBA@Cur (FNC) nanogels. The Cur content in FNC nanogels was evaluated by ultraviolet–visible spectroscopy (UV–vis) (Fig. 1C), which determined the Cur loading content to be 28 % (Fig. S1). The SEM image revealed that the nanogels had a near-spherical morphology (Fig. 1D). As shown in Fig. 1E–F, the size of FNG was around 223 nm (PDI = 0.26) determined by dynamic light scattering (DLS). Next, the storage stability of FNC at 4 °C was assessed by measuring the size and polydispersity index (PDI) of the nanogels. No significant changes in size or PDI were observed over a two-week period, indicating good short-term storage stability (Fig. 1G). Furthermore, to assess long-term stability, we extended the observation period to 28 days. The nanogels maintained consistent size and PDI throughout the storage period, confirming their colloidal stability (Fig. S2). These findings collectively validate the successful preparation and favorable colloidal stability of the FNC nanogels.

To verify the pH sensitivity of the FNC nanogels, the morphology of the nanogels under acidic conditions was observed using SEM. The results showed that the nanogels size changed in response to acidity, which simulated the stability of the nanogels at normal physiological pH and its dissociation in the acidic plaque microenvironment (Fig. 1H). Additionally, we analyzed its particle size distribution across different pH (pH = 7.4, 6.5 or 5.5). As illustrated in Fig. 1I, the particle size of the FNC nanogels increased as the solution pH decreased to 6.5, with an even more pronounced expansion observed at pH 5.5. This change is attributed to the cleavage of the Schiff base, which disrupts the nanogels structure and causes the gel to swell. Interestingly, SEM images showed a reduction in nanogel particle size under an acidic pH, while DLS measurements indicated an increase in size. This discrepancy is likely due to differences between the two methods. SEM analyzes the size of single dried particles, while DLS measures the hydrodynamic size of hydrated nanogel particles in aqueous solution, where the acidic environment may further promote their aggregation, thus leading to an apparent increase in their size value. These results suggest that FNC nanogels remain stable under normal physiological conditions but decompose in the plaque acidic microenvironment.

Given the sensitivity of nanogels to low pH, it is expected to enable the precise delivery of Cur to the plaque. To study the pH-responsive release of Cur, the drug release profile of FNC nanogels was assessed in PBS with different pHs (pH = 7.4, 6.5 or 5.5) using the dialysis method, as shown in Fig. 1J. At pH 7.4, the release rate of Cur was 29 % after 48 h. In contrast, under acidic conditions, the cumulative release increased significantly, reaching approximately 43 % at pH 5.5 after 48 h. These findings demonstrate that FNC nanogels exhibits controllable drug release capability in a simulated plaque acidic microenvironment. The hemolysis rate is a key indicator for evaluating the blood compatibility of nanogels. Therefore, different concentrations of FNC nanogels were co-incubated with fresh red cells to evaluate their blood compatibility. As shown in Fig. 1K. The hemolysis rate of FNC nanogels increased in a concentration-dependent manner. however, even at a concentration of 0.2 mg/mL, the hemolysis rate remained below 5 %. These results highlight the excellent hemocompatibility of FNC nanogels, supporting its suitability for further research applications.

### 2.2 In vitro anti-atherosclerosis effect

To confirm the safety of nanogels for subsequent *in vitro* experiments, it is essential to verify their cytotoxicity. Considering the critical role of macrophages in the progression of atherosclerosis, the viability of RAW264.7 cells treated with free Cur or FNC nanogels was assessed using the CCK-8 assay. The cells were incubated with varying concentrations of nanogels and free Cur for 24 h. As shown in Fig. S3, FNC nanogels exhibited no cytotoxic to macrophages, which demonstrated the excellent cytocompatibility of FNC nanogels.

**Fig. 2:**
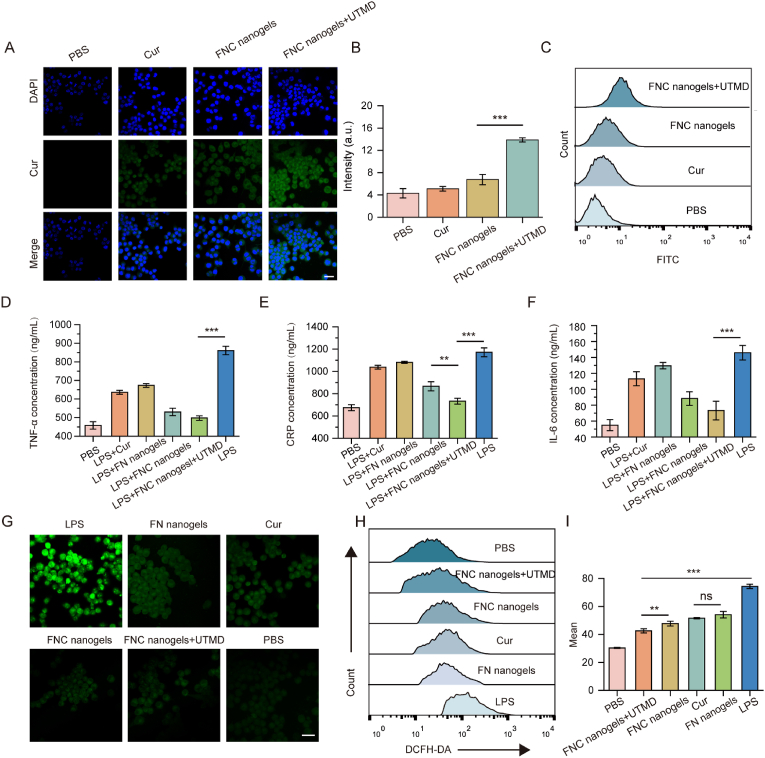
Anti-inflammatory and antioxidant effects of nanogels *in vitro*. (A–C) Cellular uptake of free Cur, FNC nanogels, and FNC nanogels combined with UTMD was evaluated using laser CLSM and flow cytometry (Scale bar = 20 μm). Means ± SD, n = 3. ∗∗∗p < 0.001, ∗p < 0.05. (D–F) Secretion levels of inflammatory factors (TNF-α, CRP, and IL-6) by RAW264.7 cells were measured under different treatments: PBS, LPS + Cur, LPS + FN nanogels, LPS + FNC nanogels, LPS + FNC nanogels + UTMD, and LPS. Means ± SD, n = 3. ∗∗∗p < 0.001, ∗∗p < 0.01. (G) Fluorescence microscopy images showing ROS generation in RAW264.7 cells under different treatments (Scale bar = 20 μm). (H–I) Flow cytometry and quantitative analysis of intracellular ROS levels in RAW264.7 cells under different treatment conditions. Means ± SD, n = 3. ∗∗∗p < 0.001, ∗∗p < 0.01, ns: no significant difference.

The uptake of therapeutic agents is a prerequisite for their efficacy. The cellular uptake of nanogels by RAW264.7 cells was analyzed using confocal laser scanning microscopy (CLSM). As shown in Fig. 2A, green fluorescence was detected in all treatment groups after 4 h of incubation, indicating successful Cur internalization. Notably, the fluorescence intensity in the FNC nanogels + UTMD group was significantly higher than that in the FNC nanogels group, suggesting that UTMD enhances cellular uptake. These observations were further validated by flow cytometry analysis, which confirmed increased nanogels uptake, with the UTMD group exhibiting a more pronounced effect (Fig. 2B–C). The agreement between CLSM and flow cytometry findings underscores the enhanced internalization mediated by UTMD. A pre-blocking experiment using free RGD peptides was performed to further investigate the cellular uptake mechanism of nanogels by macrophages. CLSM was used to visualize the internalization of FN nanogels in RAW264.7 cells, where stronger red fluorescence signals from FN (labeled with Cy5.5) were observed in the cytoplasm compared to the RGD-blocked group (Fig. S4). RGD pre-treatment significantly reduced cellular uptake compared to the non-blocked group, indicating the involvement of RGD-integrin interactions. These findings demonstrate that FN nanogels have desired macrophage-targeting capability.

Atherosclerosis is a chronic inflammatory disease of the arterial intima, with oxidative stress induced by excessive ROS playing a central role in its pathological progression. Anti-inflammatory along with the effective removal of ROS, is critical for halting atherosclerosis progression. Macrophages, integral components of the innate immune system, display remarkable plasticity, enabling them to polarize into distinct phenotypes in response to the microenvironment. They can differentiate into classically activated M1-type macrophages, which exhibit pro-inflammatory functions, or M2-type macrophages, which are linked to anti-inflammatory responses and tissue repair. The combination therapy of FN and Cur holds promise for reducing inflammatory at lesion sites, alleviating oxidative stress, and restoring homeostasis. To evaluate the *in vitro* anti-inflammatory and antioxidant potential of the FNC nanogels, LPS-activated RAW264.7 macrophages were used as a model. To assess the regulatory effect of nanogels on macrophage polarization, the expression levels of CD86 (M1 marker) and CD206 (M2 marker) were analyzed using flow cytometry. As shown in Fig. S5, LPS stimulation significantly upregulated CD86 expression, indicating a shift toward the pro-inflammatory M1 phenotype. In contrast, FNC nanogel treatment resulted in a marked reduction in CD86 expression, accompanied by an increase in CD206 level, suggesting a shift toward the anti-inflammatory M2 phenotype. Quantification of the CD206/CD86 ratio reveals that the UTMD-enhanced FNC nanogel treatment group exhibited the highest ratio among all groups, indicating the most pronounced anti-inflammatory effect. Together, these findings demonstrate that FNC nanogels promote macrophage polarization toward the M2 phenotype, supporting their potential anti-inflammatory mechanism of action. The levels of key inflammatory factors, such as tumor necrosis factor-α (TNF-α), C-reactive protein (CRP) and interleukin-6 (IL-6), were measured using ELISA kits. The results demonstrated that the inflammatory factors were up-regulated after LPS stimulation and all treatments can inhibit the expression of these inflammatory factors, with the FNC nanogels showing a more pronounced effect. Furthermore, UTMD significantly enhanced the anti-inflammatory activity of the FNC nanogels (Fig. 2D–F). These findings collectively support the potential application of FNC nanogels in modulating the inflammatory microenvironment.

ROS are key contributors to the pathogenesis of atherosclerosis. Thus, ROS levels in macrophages after treatments were evaluated by DCFH-DA staining. As shown in Fig. 2G, RAW264.7 cells stimulated with LPS exhibited significant ROS production (indicated by green fluorescence), whereas unstimulated macrophages showed minimal ROS generation. Specially, FNC nanogels demonstrated a strong inhibitory effect on ROS production, which was enhanced under UTMD. This result was further corroborated by flow cytometry analysis, which confirmed the potent antioxidant activity of FNC nanogels (Fig. 2H–I). These findings highlight the synergistic anti-inflammatory and antioxidant properties of FNC nanogels, particularly when combined with UTMD, inflammation and oxidative stress were furtherly suppressed.

### 2.3 In vivo plaque targeting and ultrasound imaging for atherosclerosis

**Fig. 3:**
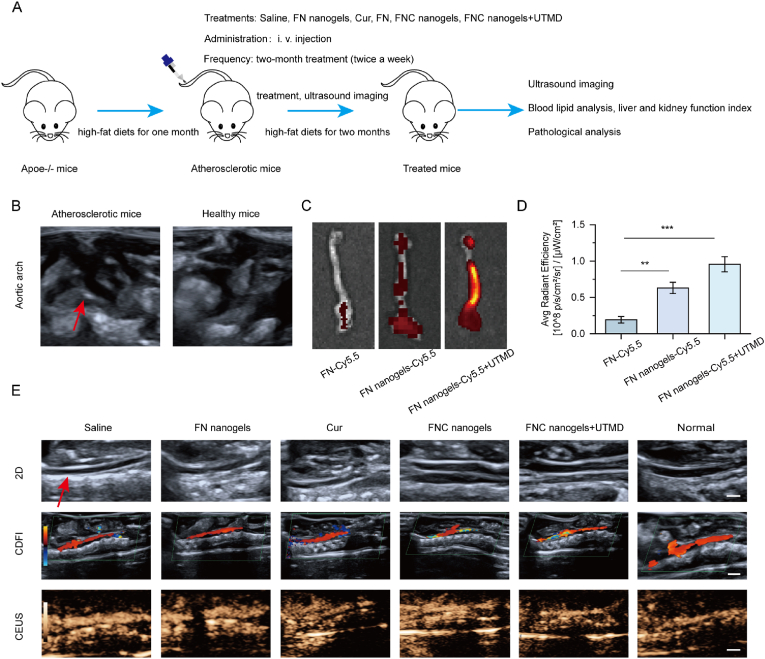
Nanogel-targeted plaque assessment and imaging evaluation. (A) Schedule of treatment protocols. (B) Ultrasound imaging of the aortic arch in early atherosclerotic mice and healthy mice to verify successful model establishment. (C, D) Fluorescence images and quantitative analysis of mean fluorescence intensity in isolated aortas 24 h after intravenous injection of free FN, FN nanogels-Cy5.5 and FN nanogels-Cy5.5 with UTMD. Means ± SD, n = 3. ∗∗∗p < 0.001, ∗∗p < 0.01. (E) Representative ultrasound images of the same mouse's abdominal aorta across three imaging modalities, acquired post-treatment in each experimental group. 2D, two-dimensional ultrasound; CDFI, color Doppler flow imaging; CEUS, contrast-enhanced ultrasound (Scale bar = 2.5 mm).

Building on the promising *in vitro* results, the therapeutic potential of FNC nanogels combined with UTMD was further evaluated *in vivo*. To induce atherosclerotic lesions, ApoE^−/−^ mice were fed a high-fat diet for four weeks to accelerate plaque formation and mimic atherosclerosis progression. Once the atherosclerosis mice were established, they were treated with saline, free Cur, FNC nanogels, or FNC nanogels combined with UTMD for two months via tail vein injection, following the treatment protocol outlined in Fig. 3A. The saline-treated ApoE^−/−^ mice served as a positive control (saline group), and the saline-treated C57BL/6 mice selected as a negative control (normal group). After one month on a high-fat diet, strong echogenic signals were observed on the inner wall of the aortic arch in ApoE^−/−^ mice, indicating successful establishment of the atherosclerosis model (Fig. 3B).

Targeted delivery to atherosclerotic plaques remains a significant challenge *in vivo*. To assess the targeting capability of the nanogels, *ex vivo* fluorescence imaging was performed 24 h after intravenous administration with Cy5.5 labeled FN, Cy5.5 labeled FN nanogels and Cy5.5 labeled FN nanogels plus UTMD in atherosclerosis mice. The results demonstrated a clear fluorescence signal from FN nanogels-Cy5.5 in the aorta, and the fluorescence signals were furtherly enhanced under UTMD, confirming effective targeting and accumulation at the atherosclerotic plaque sites (Fig. 3C–D). This property not only improves diagnostic accuracy but also enhances the therapeutic efficacy of FN nanogels for atherosclerosis.

At the end of the treatment, ultrasonography was conducted to assess atherosclerosis progression. Strong echoes were observed at the aortic arch in the saline-treated group, indicative of severe atherosclerosis. In contrast, the FNC nanogels-treated group exhibited no obvious echoes, suggesting significant alleviation of plaques. The combination of FNC nanogels and UTMD preserved normal blood flow in mouse abdominal aortas, demonstrating superior therapeutic efficacy. This effect is attributed to enhanced plaque endothelial permeability, which facilitates targeted drug delivery into atherosclerotic lesions. Notably, free Cur exhibited attenuated therapeutic effects compared to FNC nanogels, attributable to its inherently poor aqueous solubility (Fig. 3E). These findings underscore the potential of FNC nanogels, particularly in combination with UTMD, for targeted treatment of atherosclerosis.

### 2.4 Evaluation of *in vivo* anti-atherosclerosis therapy

**Fig. 4:**
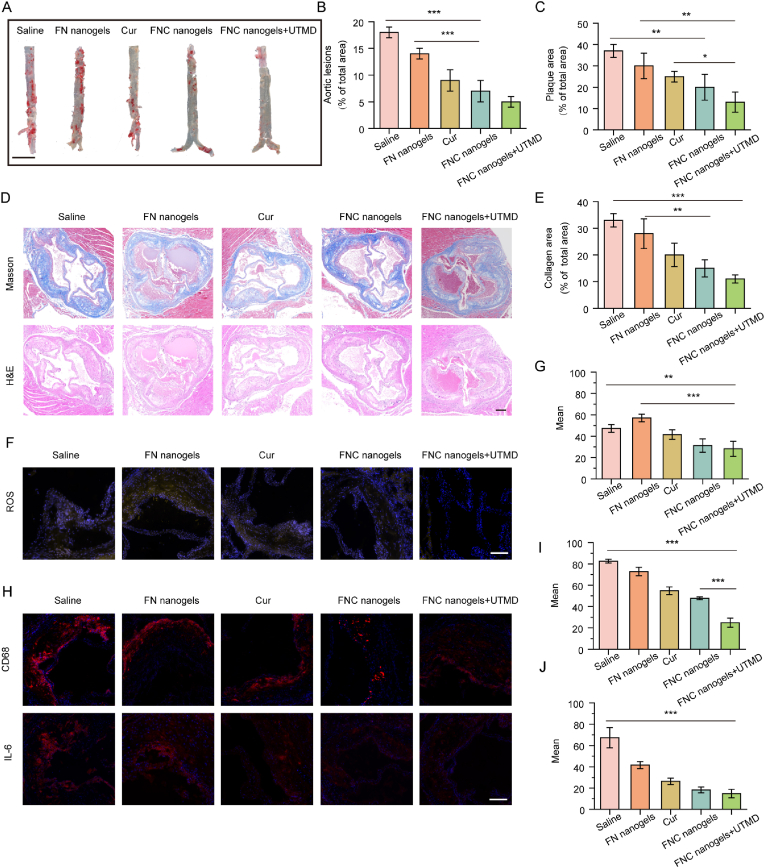
Therapeutic effects of atherosclerosis in ApoE^−/−^ mice. (A–B) Representative images of ORO-stained en face aortas and quantitative analysis of lesion area in mice after different treatments. Means ± SD, n = 3, ∗∗∗p < 0.001 (Scale bar = 5 mm). (C–E) Representative images of H&E, masson stained aortic sinus sections (Scale bar = 200 μm). The relative plaque area (C) and collagen area (E) of aortic sinus sections were quantified. Means ± SD, n = 3, ∗∗∗p < 0.001, ∗∗p < 0.01, ∗p < 0.05. (F–G) Fluorescence images of dihydroethidium (DHE) and DAPI staining of aortic sinus slices from atherosclerosis mice under different treatments and quantitative analysis of the ROS-positive areas (Yellow fluorescence represents ROS). Means ± SD, n = 3, ∗∗∗p < 0.001, ∗∗p < 0.01. (H) Immunohistochemical staining of CD68 and IL-6 in the aortic sinus region (Scale bar = 50 μm). (I–J) Quantitative analysis of the mean fluorescence of CD68^−^and IL-6-positive areas shown in (H). Means ± SD, n = 3, ∗∗∗p < 0.001, ∗∗p < 0.01.

After the treatments, the mice were euthanized, and their aortas were isolated, opened longitudinally, and stained with Oil Red O (ORO) to visualize atherosclerotic plaques. Red-stained areas, representing plaque regions, were quantified for analysis (Fig. 4A–B). Aortas from the saline-treated group exhibited extensive atherosclerotic lesions. While the Cur group showed a modest reduction in atherosclerosis progression compared to the control group, the therapeutic effect was limited, likely due to the poor bioavailability of Cur. In contrast, FNC nanogels, leveraging its plaque-targeting capability, significantly reduced the aortic atherosclerosis lesion area. The ORO-stained regions were markedly smaller, and the therapeutic efficacy was further enhanced when FNC nanogels were combined with UTMD. These findings highlight the superior potential of FNC nanogels for mitigating atherosclerosis, particularly in combination with UTMD.

Hematoxylin and eosin (H&E) staining and Masson staining were conducted on aortic sinus sections to furtherly evaluate luminal stenosis rates and collagen content within plaques. As shown in Fig. 4C–E, FNC nanogels significantly reduced luminal stenosis rates in the aortic sinus compared to the saline group, with minimal collagen infiltration observed in the plaques. These findings indicate that FNC nanogels, particularly when combined with UTMD, effectively mitigates the progression of atherosclerosis in ApoE^−/−^ mice, demonstrating superior efficacy compared to free Cur.

To elucidate the mechanism of FNC nanogels treatment for atherosclerosis *in vivo*, frozen sections of the aortic sinus from atherosclerotic mice were prepared and stained with anti-CD68, anti-IL-6, and anti-ROS antibodies to evaluate inflammation and ROS levels. As shown in Fig. 4F–G, yellow fluorescence indicates ROS presence. The saline-treated group exhibited strong fluorescence intensity, indicating elevated oxidative stress in the aortic sinus. In contrast, weaker fluorescence was observed in the FNC nanogels and FNC nanogels + UTMD groups, highlighting the superior ROS-scavenging ability of FNC nanogels compared to free Cur. These results demonstrate the potential of FNC nanogels in reducing oxidative stress and mitigating atherosclerosis progression.

Macrophages and their secretion of inflammatory factors play a critical role in the progression of atherosclerosis. To assess the inflammatory response, macrophage content within plaques was analyzed using CD68 and IL-6 as representative markers. As shown in Fig. 4H–J, red staining highlights the presence of positive macrophages. Plaques in the FNC nanogels and FNC nanogels + UTMD groups exhibited significantly reduced CD68 and IL-6 staining compared to the saline-treated group, demonstrating the anti-inflammatory efficacy of FNC nanogels, which was further enhanced by its combination with UTMD.

The FNC nanogels developed in this study demonstrated excellent anti-inflammatory effects by enabling targeted delivery to macrophages and promoting their polarization toward the M2 phenotype. While clinically used anti-inflammatory agents like statins exhibit some anti-inflammatory and antioxidant properties, their therapeutic efficacy is often limited by systemic side effects and a lack of targeting capability. It is important to note that nanodrug systems primarily function through localized delivery, whereas statins rely on systemic distribution for their effects [35].

### 2.5 Biosafety assessment

**Fig. 5:**
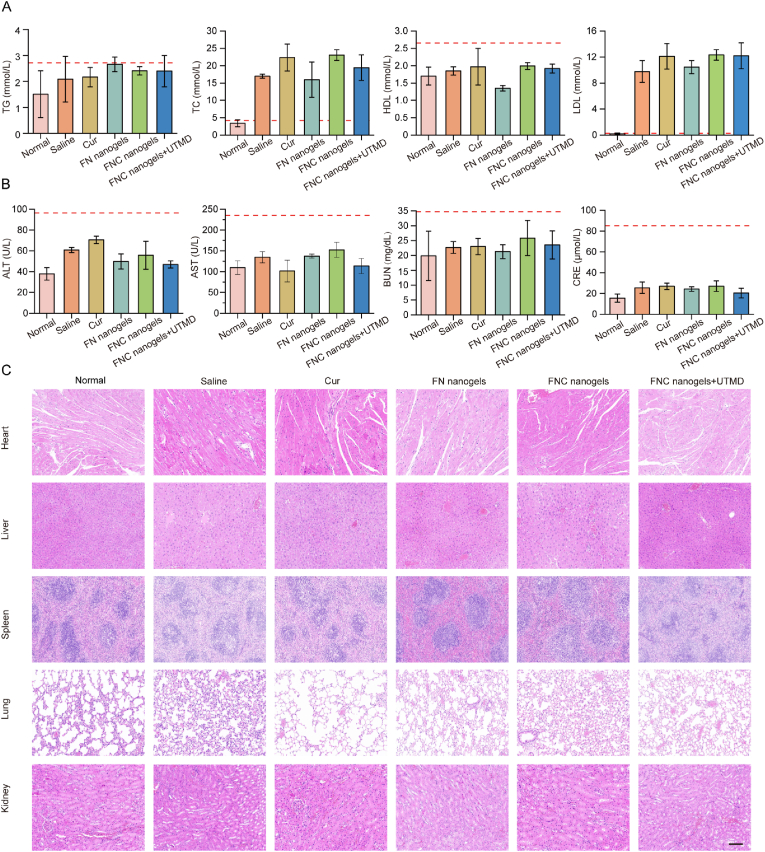
Serological analysis and safety evaluation. (A) Serum levels of triglycerides (TG), total cholesterol (TC), high-density lipoprotein (HDL), and low-density lipoprotein (LDL) in ApoE^−/−^ mice across different treatment groups. Means ± SD, n = 3. (B) Biochemical indices of liver and kidney function in atherosclerotic mice following various treatments. Means ± SD, n = 3. (C) H&E-stained sections of major organs collected from mice at the conclusion of the treatment period for each group (Scale bar = 150 μm).

Lipid metabolism is crucial in the onset and progression of atherosclerosis. Common atherosclerosis models, such as ApoE^−/−^ mice, exhibit significant lipid metabolic disturbances due to genetic deficiency. To assess this, we measured the serum lipid profiles at the end of treatment. The results showed that, compared to the negative control group (normal group), a high-fat diet significantly elevated TC and LDL levels in atherosclerotic mice. The positive control group (saline group) also exhibited elevated TC and LDL levels compared to the normal group, which should be due to the lack of therapeutic intervention against high-fat diet-induced lipid abnormalities. However, no significant differences were observed in lipid parameters (TC, TG, LDL, and HDL) among the treated groups (Fig. 5A). These findings suggest that the anti-atherosclerotic effects of FNC nanogels are mediated through their anti-inflammatory and antioxidant properties rather than through modulation of lipid metabolism.

To evaluate the biosafety of FNC nanogels, major organs (heart, liver, spleen, lungs, and kidneys) were collected from treated mice for histological analysis, and serum samples were assessed for liver and kidney function markers. Clinical biochemical analyses revealed that serum levels of alanine aminotransferase (ALT), aspartate aminotransferase (AST), blood urea nitrogen (BUN), and creatinine (CRE) remained within normal reference ranges (Fig. 5B), indicating no significant impact on liver or kidney function. Histological examination using H&E staining showed no signs of tissue necrosis, abnormal cell morphology, or inflammatory cell infiltration in any treatment group compared to C57BL/6 mice (Fig. 5C). In addition, the mice exhibited consistent weight gain during the treatment period (Fig. S6), further supporting the good systemic tolerance of the formulation.

To further assess the biocompatibility of FNC nanogels, healthy C57BL/6 mice were injected intravenously with FNC nanogels twice a week for a total of four injections, and observed for 14 days. Histopathological examination of major organs (H&E staining) revealed no abnormalities, confirming the absence of organ toxicity (Fig. S7) and supporting their potential as a safe and promising nanodelivery system for therapeutic applications. Moreover, the FNC nanogels are made from readily available materials and can be synthesized using a simple, scalable method, which enhances their potential for clinical translation. With demonstrated excellent anti-inflammatory and antioxidant performance in both *in vitro* and *in vivo* studies, along with favorable stability and biosafety profiles, we believe that the FNC nanogels hold significant promise for clinical translation.

## 3 Conclusion

In summary, we developed the pH-responsive FN nanogels loaded with Cur, utilizing the natural RGD sequence on FN to specifically target macrophages in atherosclerotic plaques. Enhanced by UTMD technology, the nanogels demonstrated increased accumulation and deeper penetration into plaque regions. Within the plaque microenvironment, the pH-sensitive Schiff base bond was cleaved, causing the nanogels to disintegrate and release its active components. This process resulted in pronounced anti-inflammatory and antioxidant effects, effectively slowing the progression of atherosclerosis. This FN-based gelation approach presents a promising therapeutic strategy for treating atherosclerosis.

## CRediT authorship contribution statement

**Mengjiao Zhang:** Writing – original draft, Investigation, Methodology. **Xianghao Xiao:** Methodology, Writing – original draft, Investigation. **Xinyi Li:** Data curation. **Xiangyang Shi:** Writing – review & editing, Supervision. **Zhaojun Li:** Supervision, Writing – review & editing, Funding acquisition.

## Ethics approval and consent to participate

All animal experiments were conducted with the approval of the Animal Care and Use Committee of Donghua University and in compliance with the guidelines of the National Institute of Health (China).

## Funding

This work was supported by the 10.13039/501100001809National Natural Science Foundation of China (82472008); the 10.13039/501100014175Shanghai Health and Family Planning Commission Fund (202240235); the 10.13039/100007219Natural Science Foundation of Shanghai (21ZR1451400); and the Technology Innovation Project of the Jiading Branch of 10.13039/501100013103Shanghai General Hospital (2024-KJJB-02, 2024-KJJB-03, and 2025-KJ-QN-05).

## Declaration of competing interest

The authors declare that they have no known competing financial interests or personal relationships that could have appeared to influence the work reported in this paper.

## Data Availability

Data will be made available on request.

## References

[1] Björkegren J.L.M., Lusis A.J. (2022). Atherosclerosis: recent developments. Cell.

[2] Li Y., Che J., Chang L., Guo M., Bao X., Mu D., Sun X., Zhang X., Lu W., Xie J. (2022). CD47- and integrin α4/β1-comodified-macrophage-membrane-coated nanoparticles enable delivery of colchicine to atherosclerotic plaque. Adv. Healthcare Mater..

[3] Roeters van Lennep J.E., Tokgözoğlu L.S., Badimon L., Dumanski S.M., Gulati M., Hess C.N., Holven K.B., Kavousi M., Kayıkçıoğlu M., Lutgens E., Michos E.D., Prescott E., Stock J.K., Tybjaerg-Hansen A., Wermer M.J.H., Benn M. (2023). Women, lipids, and atherosclerotic cardiovascular disease: a call to action from the European Atherosclerosis Society. Eur. Heart J..

[4] Tedgui A., Mallat Z. (2006). Cytokines in atherosclerosis: pathogenic and regulatory pathways. Physiol. Rev..

[5] Mizuno Y., Jacob R.F., Mason R.P. (2011). Inflammation and the development of atherosclerosis. J. Atheroscler. Thromb..

[6] Yuan T., Yang T., Chen H., Fu D., Hu Y., Wang J., Yuan Q., Yu H., Xu W., Xie X. (2019). New insights into oxidative stress and inflammation during diabetes mellitus-accelerated atherosclerosis. Redox Biol..

[7] Eid A.H., Pintus G., Yazbi A.E., Orekhov A., Parenti A., Assaf R., Aramouni K., Shaito A. (2022). Oxidative stress-induced endothelial dysfunction in cardiovascular diseases. Front. Biosci.-Landmark.

[8] Malekmohammad K., Sewell R.D.E., Rafieian-Kopaei M. (2019). Antioxidants and atherosclerosis: mechanistic aspects. Biomolecules.

[9] Khatana C., Saini N.K., Chakrabarti S., Saini V., Sharma A., Saini R.V., Saini A.K. (2020). Mechanistic insights into the oxidized low-density lipoprotein-induced atherosclerosis. Oxid. Med. Cell. Longevity.

[10] Chmielowski R.A., Abdelhamid D.S., Faig J.J., Petersen L.K., Gardner C.R., Uhrich K.E., Joseph L.B., Moghe P.V. (2017). Athero-inflammatory nanotherapeutics: ferulic acid-based poly(anhydride-ester) nanoparticles attenuate foam cell formation by regulating macrophage lipogenesis and reactive oxygen species generation. Acta Biomater..

[11] Gao A., Gupta S., Shi H., Liu Y., Schroder A.L., Witting P.K., Ahmad G. (2021). Pro-inflammatory serum amyloid a stimulates renal dysfunction and enhances atherosclerosis in Apo E-deficient mice. Int. J. Mol. Sci..

[12] Kong P., Cui Z.Y., Huang X.F., Zhang D.D., Guo R.J., Han M. (2022). Inflammation and atherosclerosis: signaling pathways and therapeutic intervention. Signal Transduction Targeted Ther..

[13] Dong Z.M., Chapman S.M., Brown A.A., Frenette P.S., Hynes R.O., Wagner D.D. (1998). The combined role of P- and E-selectins in atherosclerosis. J. Clin. Investig..

[14] Masana L., Plana N., Andreychuk N., Ibarretxe D. (2023). Lipid lowering combination therapy: from prevention to atherosclerosis plaque treatment. Pharmacol. Res..

[15] Ruoslahti E., Pierschbacher M., Engvall E., Oldberg Å., Hayman E.G. (1982). Molecular and biological interactions of fibronectin. J. Invest. Dermatol..

[16] Huet-Calderwood C., Rivera-Molina F.E., Toomre D.K., Calderwood D.A. (2023). Fibroblasts secrete fibronectin under lamellipodia in a microtubule- and myosin II–dependent fashion. J. Cell Biol..

[17] Hershkoviz R., Alon R., Gilat D., Lider O. (1992). Activated T lymphocytes and macrophages secrete fibronectin which strongly supports cell adhesion. Cell. Immunol..

[18] Rieder H., Ramadori G., Dienes H.P., Meyer zum Büschenfelde K.H. (1987). Sinusoidal endothelial cells from Guinea pig liver synthesize and secrete cellular fibronectin in vitro. Hepatology.

[19] Wu C., Weis S.M., Cheresh D.A. (2023). Upregulation of fibronectin and its integrin receptors - an adaptation to isolation stress that facilitates tumor initiation. J. Cell Sci..

[20] Labat-Robert J. (2012). Cell-matrix interactions, the role of fibronectin and integrins. A survey. Pathol. Biol..

[21] Ruoslahti E., Pierschbacher M.D. (1987). New perspectives in cell adhesion: RGD and integrins. Science.

[22] Zhou N., Ma X., Hu W., Ren P., Zhao Y., Zhang T. (2021). Effect of RGD content in poly(ethylene glycol)-crosslinked poly(methyl vinyl ether-alt-maleic acid) hydrogels on the expansion of ovarian cancer stem-like cells. Mater. Sci. Eng., C.

[23] Lin X., Sun Y., Yang S., Yu M., Pan L., Yang J., Yang J., Shao Q., Liu J., Liu Y., Zhou Y., Wang Z. (2021). Omentin-1 modulates macrophage function via integrin receptors αvβ3 and αvβ5 and reverses plaque vulnerability in animal models of atherosclerosis. Front. Cardiovasc. Med..

[24] Gao Y., Dai W., Ouyang Z., Shen M., Shi X. (2023). Dendrimer-mediated intracellular delivery of fibronectin guides macrophage polarization to alleviate acute lung injury. Biomacromolecules.

[25] Dai W., Zhan M., Gao Y., Sun H., Zou Y., Laurent R., Mignani S., Majoral J.P., Shen M., Shi X. (2024). Brain delivery of fibronectin through bioactive phosphorous dendrimers for Parkinson's disease treatment via cooperative modulation of microglia. Bioact. Mater..

[26] Zhan M., Sun H., Wang Z., Li G., Yang R., Mignani S., Majoral J.P., Shen M., Shi X. (2024). Nanoparticle-mediated multiple modulation of bone microenvironment to tackle osteoarthritis. ACS Nano.

[27] Sun H., Zhan M., Karpus A., Zou Y., Li J., Mignani S., Majoral J.P., Shi X., Shen M. (2024). Bioactive phosphorus dendrimers as a universal protein delivery system for enhanced anti-inflammation therapy. ACS Nano.

[28] Patel S.S., Acharya A., Ray R.S., Agrawal R., Raghuwanshi R., Jain P. (2020). Cellular and molecular mechanisms of curcumin in prevention and treatment of disease. Crit. Rev. Food Sci. Nutr..

[29] Lin D., Xiao L., Qin W., Loy D.A., Wu Z., Chen H., Zhang Q. (2022). Preparation, characterization and antioxidant properties of curcumin encapsulated chitosan/lignosulfonate micelles. Carbohydr. Polym..

[30] Pontes-Quero G.M., Benito-Garzón L., Pérez Cano J., Aguilar M.R., Vázquez-Lasa B. (2021). Amphiphilic polymeric nanoparticles encapsulating curcumin: Antioxidant, anti-inflammatory and biocompatibility studies. Mater. Sci. Eng., C.

[31] Luo X., Zhang J., Shao S., Wu R., Du L., Yuan J., Li Z. (2019). Improving ultrasound gene transfection efficiency in vitro. Advanced Ultrasound in Diagnosis and Therapy.

[32] Li H., Zhang Y., Shu H., Lv W., Su C., Nie F. (2022). Highlights in ultrasound-targeted microbubble destruction-mediated gene/drug delivery strategy for treatment of malignancies. Int. J. Pharm..

[33] Yang L., Chen L., Fang Y., Ma S. (2021). Downregulation of GSK-3β expression via ultrasound-targeted microbubble destruction enhances atherosclerotic plaque stability in New Zealand rabbits. Ultrasound Med. Biol..

[34] Zhou J., Niu C., Huang B., Chen S., Yu C., Cao S., Pei W., Guo R. (2022). Platelet membrane biomimetic nanoparticles combined with UTMD to improve the stability of atherosclerotic plaques. Front. Chem..

[35] Yang F., Xue J., Wang G., Diao Q. (2022). Nanoparticle-based drug delivery systems for the treatment of cardiovascular diseases. Front. Pharmacol..

