# Supplementary material for: pH-sensitive fibronectin nanogels combined with UTMD for anti-atherosclerosis treatment through anti-inflammatory and antioxidant effects
Source: Mater Today Bio. 2025 Jul 8;33:102044. doi: 10.1016/j.mtbio.2025.102044 (PMC12274839; doi:10.1016/j.mtbio.2025.102044)
Supplement: Multimedia component 1 [file mmc1.doc]

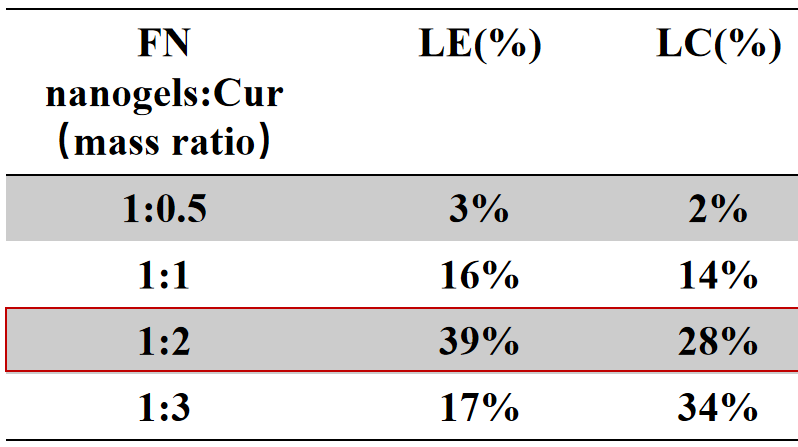


**Fig. S1.** Drug loading and encapsulation efficiency of FNC nanogels at various mass ratios.


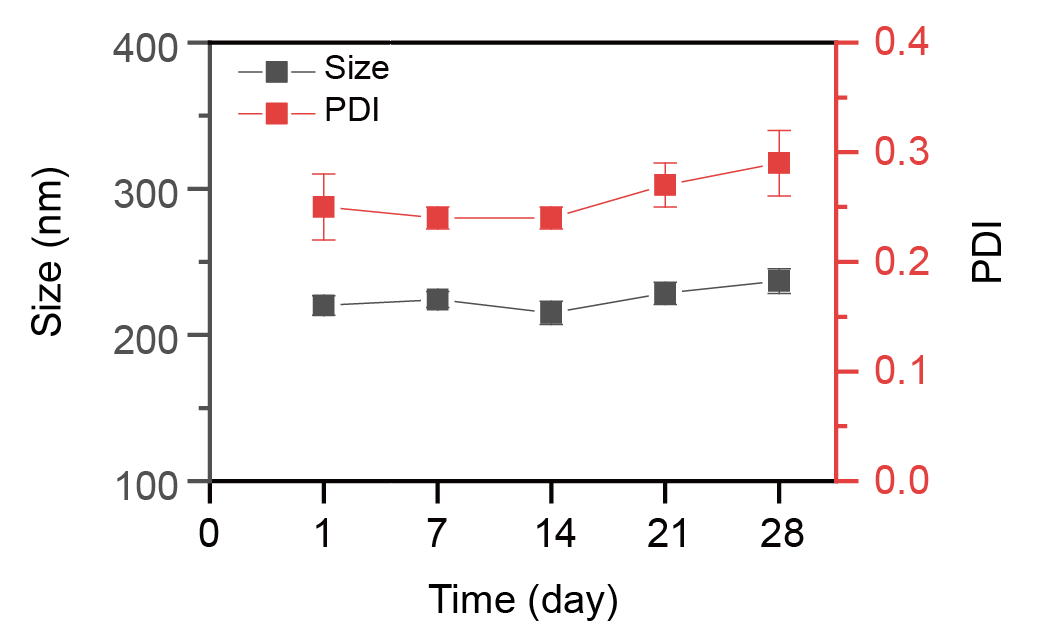


**Fig. S2.** DLS analysis of FNC nanogels stored at 4 °C for 28 days. Means ± SD, n = 3.


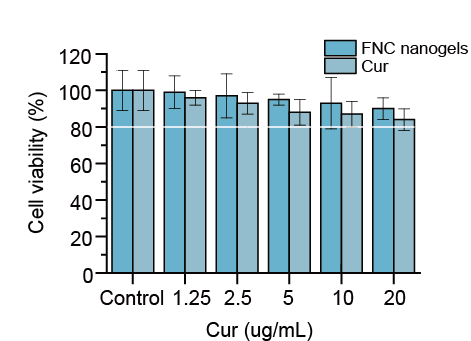


**Fig. S3.** Cell viability of RAW264.7 cells after a 24-h incubation with various concentrations of free Cur or FNC nanogels. Means ± SD, n = 3.


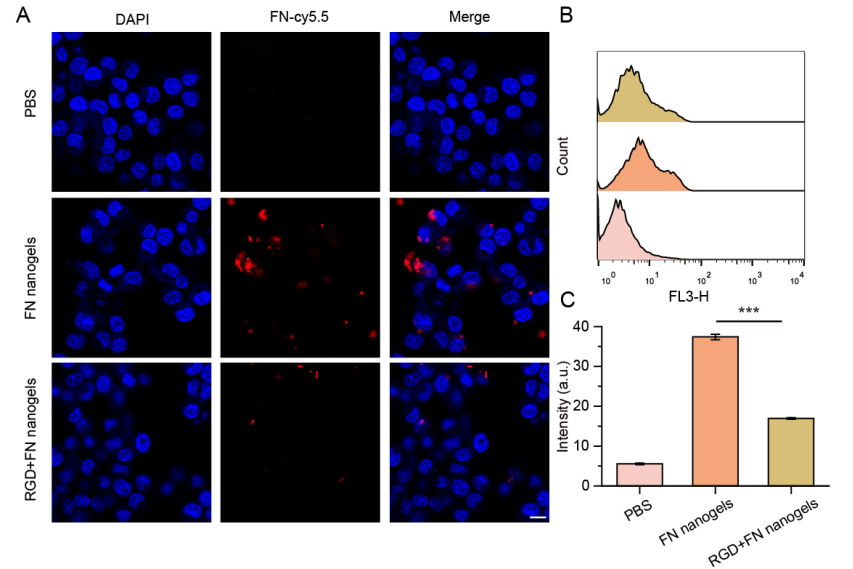


**Fig. S4.** (A) Confocal microscopy images and (B) Flow cytometric histograms and (C) corresponding fluorescence intensity of RAW264.7 cells treated with PBS or Cy5.5-labeled FN nanogels, with or without RGD pre-treatment (Scale bar = 20 μm).


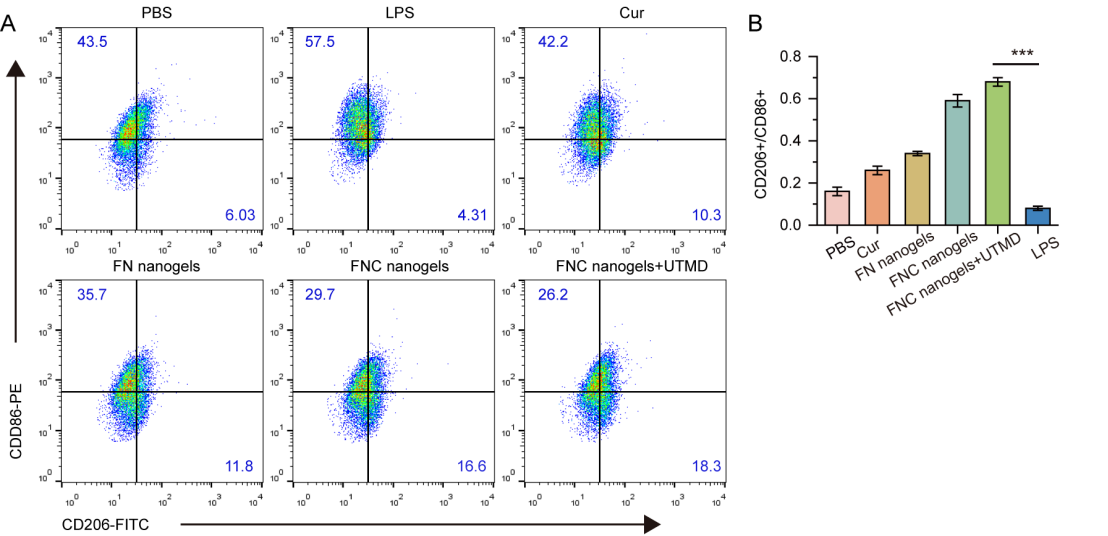


**Fig. S5. (A)** Flow cytometry analysis of CD206 and CD86 expression on RAW264.7 cells following 24 h treatment with different formulations. **(B)** Quantification of CD206/CD86 expression ratio on RAW264.7 cells after 24 h treatment with experimental formulations (n = 3).


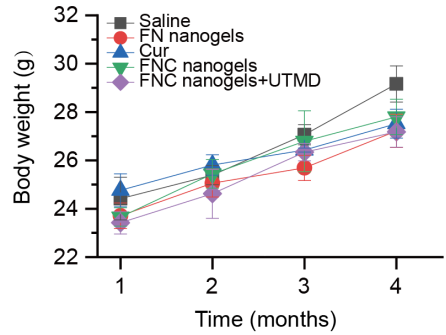


**Fig. S6.** Body weight changes in atherosclerotic mice during treatment. The results show that the nanogels had no significant impact on the body weight of mice, indicating good biosafety. Means ± SD, n = 3.


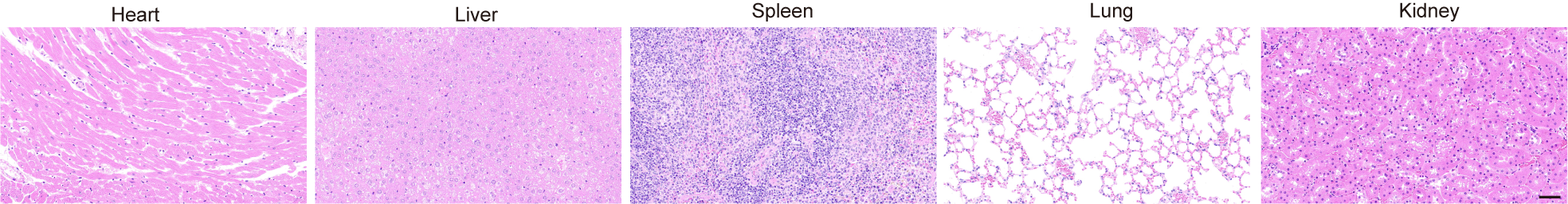


**Fig. S7.** H&E staining of heart, liver, spleen, lungs, and kidneys from healthy mice 14 days after injection of the nanogels (Scale bar = 50 μm).
